# Circulating microRNA, secreted microRNA and exogenous plant microRNA

**DOI:** 10.1186/1479-5876-10-S2-A15

**Published:** 2012-10-17

**Authors:** Chenyu Zhang

**Affiliations:** 1 School of Life Sciences, Nanjing University, 210093, China

## 

Dysregulated expression of microRNAs (miRNAs) in various tissues has been associated with a variety of diseases, including cancers. Here we demonstrate that miRNAs are present in the serum and plasma of humans and other animals such as mice, rats, bovine fetuses, calves and horses. The levels of miRNAs in serum are stable, reproducible, and consistent among individuals of the same species. Employing Solexa, we sequenced all serum miRNAs of healthy Chinese subjects and found over 100 and 91 serum miRNAs in male and female subjects, respectively. We also identified specific expression patterns of serum miRNAs for lung cancer, colorectal cancer and diabetes, providing evidence that serum miRNAs contain fingerprints for various diseases. Through these analyses, we conclude that serum miRNAs can serve as potential biomarkers for the detection of various cancers and other diseases.

Here, we also report that secreted miRNAs can serve as novel signaling molecules mediating intercellular communication. In human blood cells and cultured THP-1 cells, miR-150 was selectively packaged into microvesicles (MVs) and actively secreted. THP-1–derived MVs rapidly entered and delivered miR-150 into human microvascular endothelial cells (HMEC-1), and elevated exogenous miR-150 delivered from MVs effectively reduced c-Myb expression and enhanced cell migration. In vivo studies confirmed that intravenous injection of THP-1 MVs significantly increased the level of miR-150 in mouse blood vessels. MVs isolated from the plasma of patients with atherosclerosis contained higher levels of miR-150, and they more effectively promoted HMEC-1 cell migration than MVs from healthy donors. These results demonstrate that cells actively secrete miRNAs and deliver them into specific recipient cells where the exogenous miRNAs can regulate target gene expression and recipient cell function.

